# Whole‐Exome Sequencing Reveals the Genetic Landscape and Genotype–Phenotype Spectrum of Dystonia in a Chinese Cohort

**DOI:** 10.1002/cns.71203

**Published:** 2026-10-11

**Authors:** Yiqing Gong, Huirong Peng, Zhao Chen, Jiawei He, Linliu Peng, Qian Jiang, Chunrong Wang, Linlin Wan, Lang He, Yuting Shi, Rong Qiu, Hong Jiang

**Affiliations:** ^1^ Department of Neurology, Xiangya Hospital Central South University Changsha Hunan China; ^2^ Key Laboratory of Hunan Province in Neurodegenerative Disorders Central South University Changsha Hunan China; ^3^ Bioinformatics Center and National Clinical Research Center for Geriatric Disorders, Xiangya Hospital Central South University Changsha Hunan China; ^4^ Hunan International Scientific and Technological Cooperation Base of Neurodegenerative and Neurogenetic Diseases Changsha Hunan China; ^5^ Department of Pathology, Xiangya Hospital Central South University Changsha Hunan China; ^6^ Department of Radiology, Xiangya Hospital Central South University Changsha Hunan China; ^7^ National International Collaborative Research Center for Medical Metabolomics Central South University Changsha Hunan China; ^8^ School of Computer Science and Engineering Central South University Changsha Hunan China; ^9^ Department of Neurology The Third Xiangya Hospital of Central South University Changsha Hunan China; ^10^ Furong Laboratory Central South University Changsha Hunan China; ^11^ Brain Research Center Central South University Changsha Hunan China

**Keywords:** Chinese cohort, dystonia, genetic landscape, genotype–phenotype spectrum, molecular diagnosis, whole‐exome sequencing

## Abstract

**Aims:**

To systematically characterize the genetic landscape of dystonia in a Mainland Chinese cohort, where over 60% of patients currently lack a molecular diagnosis due to limited large‐scale genomic studies.

**Methods:**

Whole‐exome sequencing (WES) was performed in a cohort of 147 clinically diagnosed dystonia patients, with variants prioritized by population frequency, CADD/REVEL scores, and ACMG criteria; repeat expansion screening was also conducted to evaluate potential repeat‐expansion phenocopies.

**Results:**

The overall diagnostic yield was 33.3%, with significantly higher rates in generalized and childhood‐onset dystonia patients. We expanded the mutational and phenotypic spectra of established dystonia genes, including *ATP1A3*, *KMT2B*, *GCH1*, and *TOR1A*. Repeat expansion screening identified *ATXN8OS* and *TBP* expansions of uncertain clinical significance. Notably, two heterozygous *FIG4* variants (c.1038_1039del and c.2181‐2A>G) were identified as candidate findings requiring further validation. Exploratory HEK‐293 T cell analyses showed that the frameshift variant produced a truncated protein and was associated with lysosome‐related cellular alterations and reduced cell viability.

**Conclusion:**

This study delineates the genetic architecture and genotype–phenotype spectrum of dystonia in a Chinese cohort, providing population‐specific evidence for the molecular classification and genetic evaluation of dystonia.

## Graphical abstract

Whole‐exome sequencing of 147 Chinese patients with dystonia revealed an overall molecular diagnostic yield of 33.3%, with higher yields in generalized and childhood‐onset cases. The study expands the genotype–phenotype spectrum of established dystonia genes and provides population‐specific evidence to support genetic evaluation and molecular classification.

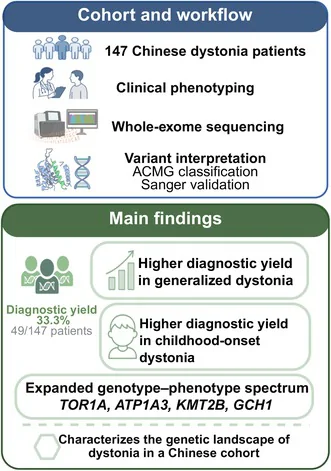

## 1 Introduction

Dystonia is a clinically and genetically heterogeneous movement disorder characterized by sustained or intermittent abnormal movements, postures, or both, which are typically patterned and repetitive and may be tremulous or jerky [1]. Its phenotypic spectrum ranges from focal manifestations, such as neck, eye, limb, or craniofacial involvement, to generalized forms that may include dysphagia or speech deficits [2, 3]. Although dystonia is primarily diagnosed based on clinical evaluation, its etiology involves complex genetic and environmental interactions [4]. To date, more than 30 known genes, including *TOR1A*, *GCH1*, *ANO3*, *KMT2B*, and *VPS16*, have been associated with dystonia [5, 6]. Nevertheless, a substantial proportion of patients remain without molecular diagnoses, reflecting both unidentified genetic factors and limitations of current testing approaches.

Whole‐exome sequencing (WES) has advanced the investigation of the genetic basis of dystonia. Compared with traditional gene‐panel or Sanger sequencing, WES enables comprehensive analysis of coding regions, allowing high‐resolution detection of single‐nucleotide variants and small insertions or deletions [7]. Several studies have demonstrated the diagnostic utility of WES in identifying dystonia‐associated variants [8, 9, 10]. For example, a European multicenter study reported pathogenic or likely pathogenic (P/LP) variants in 19% of sporadic dystonia cases, including *IMPDH2* as a candidate gene in neurodevelopmental syndromes with dystonia [11]. Other cohorts focusing on early‐onset generalized dystonia have revealed associations with *GCH1*, *THAP1*, *TOR1A*, *ANO3*, and *ADCY5* [12]. Despite these advances, large‐scale WES studies in Mainland Chinese dystonia cohorts remain limited, representing a critical knowledge gap given population‐specific genetic backgrounds. Pathophysiologically, dystonia involves dysfunction of basal ganglia–cerebello–thalamo–cortical networks, which may contribute to its clinical overlap with other movement disorders [13]. Combined dystonia may co‐occur with features such as ataxia and cognitive impairment, complicating differentiation from other neurological conditions. In selected atypical presentations, phenotypic overlap with repeat‐expansion disorders, including Huntington's disease and spinocerebellar ataxias, may also be considered during genetic evaluation [14, 15].

In this study, we performed WES and repeat‐expansion screening in a cohort of dystonia patients from Mainland China, aiming to characterize the genetic landscape, refine genotype–phenotype correlations, and explore population‐relevant genetic patterns.

## 2 Methods

### 2.1 Participants

This study enrolled 147 patients with dystonia who were consecutively recruited from the Department of Neurology, Xiangya Hospital, Central South University, between January 2020 and January 2026. Written informed consent was obtained from all participants or their legal guardians before enrollment. All patients met the updated consensus‐based clinical diagnostic criteria for dystonia [1] and were independently diagnosed by two experienced neurologists. Patients with a history of prior genetic testing were excluded from the study. Acquired etiologies were systematically ruled out through brain magnetic resonance imaging (MRI) and other relevant clinical evaluations. For each proband and available family members, 5 mL of peripheral venous blood was collected, and genomic DNA was extracted using the standard phenol‐chloroform method.

### 2.2 Clinical Assessments

Comprehensive clinical data were collected, including demographic information, age at onset, disease duration, and dystonia subtype. Age at onset was categorized into five groups: infancy (< 2 years), childhood (2–12 years), adolescence (13–18 years), early adulthood (19–40 years), and late adulthood (over 40 years). Based on dystonia distribution, patients were classified into five categories: generalized, multifocal, focal, segmental, and hemidystonia. Furthermore, dystonia was classified as either isolated or combined, depending on the presence of other coexisting movement disorders such as tremor or ataxia. Genetic movement disorder syndromes were described using gene‐based nomenclature according to the International Parkinson and Movement Disorder Society Task Force recommendations [16].

### 2.3 Ethics

The study was approved by the Ethics Committee of Xiangya Hospital, Central South University, China (Reference number: 202310206), and all participants provided written informed consent in accordance with the Declaration of Helsinki.

### 2.4 WES and In Silico Analysis

Genomic DNA samples were sequenced using the Illumina NovaSeq high‐throughput platform. The raw sequencing data were first subjected to quality control using Illumina Sequence Control Software (SCS) and then processed for further analysis [17].

Sequence alignment was performed using BWA‐MEM (v0.7.17) to align the data to the human reference genome (GRCh37). Variant calling was performed using the GATK Best Practices pipeline (v4.2). Variants were filtered using a three‐tiered strategy to identify potentially pathogenic variants: (1) variants with a minor allele frequency (MAF) ≥ 1% were excluded based on population databases such as ExAC, the 1000 Genomes Project, and gnomAD (v2.1.0) [18, 19]; (2) functional prediction: assessing the impact of variants was predicted using the combined annotation dependent depletion (CADD) score (≥ 20) and the REVEL score (≥ 0.65) [20, 21]; and (3) clinical annotation: variants were classified according to the ACMG 2015 guidelines, with a focus on pathogenic and likely pathogenic variants [22, 23]. All candidate variants were validated by Sanger sequencing. Sequence alignment analysis was conducted using Chromas (v2.6.6) and Mutation Surveyor (v5.0.1). For significant P/LP mutations, cosegregation analysis was performed on family samples to investigate inheritance patterns and their association with disease. We utilized AlphaFold3 for protein modeling and employed Pymol for visualizing 3D protein structures.

### 2.5 Repeat Expansion Screening

Short tandem repeat (STR) genotyping was performed on BAM files using Expansion Hunter (v5.0.0) and REViewer (v0.2.7) [24, 25]. The analysis included 9 repeat expansion disorders, including spinocerebellar ataxia (SCA 1, 2, 3, 6, 7, 17), dentatorubral‐pallidoluysian atrophy (DRPLA), Huntington's disease, spinal and bulbar muscular atrophy (Kennedy's disease). For repeat‐expansion disorders located in non‐coding regions, including SCA12, SCA36, DM1, and DM2, negative calls derived from WES data were considered technically inconclusive rather than evidence for the definitive absence of repeat expansion, because these loci are typically poorly captured by exome sequencing. Putative positive repeat‐expansion findings were independently confirmed using fluorescent capillary electrophoresis.

### 2.6 Copy Number Variation (CNV) Analysis

CNVs were analyzed using a read‐depth‐based approach, with per‐interval coverage calculated using Bedtools (v2.30.0), followed by comparison of each sample against a baseline constructed from samples processed with the same capture kit. Candidate exonic deletions and duplications were visually inspected and validated by multiplex ligation‐dependent probe amplification (MLPA) when DNA samples were available.

### 2.7 Cell Experiment Procedures

HEK‐293T cells were cultured in 10% DMEM medium and transfected with wild‐type (WT) or mutant (MUT) *FIG4* plasmids (constructed by GenScript Biotech) using Lipofectamine 3000 reagent (Thermo Fisher Scientific). For protein analysis, western blotting was performed using the following antibodies: anti‐FLAG (20543, Proteintech), anti‐LC3 (3868T, Cell Signaling Technology), anti‐p62 (P0067, Sigma‐Aldrich), anti‐LAMP1 (9091S, Cell Signaling Technology), and anti‐GAPDH (10,494, Proteintech).

To characterize the functional effects of the candidate *FIG4* variants, expression constructs were generated for m1 and m2. The m1 construct contained the c.1038_1039del variant, corresponding to p.L347Gfs*15. For the splice‐site variant c.2181‐2A>G, a minigene splicing reporter containing the relevant *FIG4* genomic region encompassing the exon 19–intron 19‐20–exon 20 junction was constructed. WT and MUT minigene plasmids, which differed only at the c.2181‐2A>G position, were transfected into HEK‐293T cells. Total RNA was extracted and reverse‐transcribed, and the minigene‐derived transcripts were amplified by PCR and subjected to Sanger sequencing to determine the resulting splice junction. The m2 expression construct was generated based on the experimentally defined aberrant transcript, which retained one terminal intronic G and was predicted to result in p.N728Kfs*5, and encoded the corresponding N‐terminal FLAG‐tagged truncated *FIG4* protein. All constructs were sequence‐verified before transfection.

To evaluate lysosomal function, live cells were stained with 200 nM LysoTracker Red (L7528, Thermo Fisher Scientific) and imaged by confocal microscopy, while parallel samples were trypsinized, PBS‐resuspended, and analyzed by FACS Canto II flow cytometer (BD Biosciences) with FlowJo software (v10.8.1) for quantitative assessment. LysoTracker Red was used as an acidotropic marker of LysoTracker‐positive acidic compartments. Additionally, cells were fixed in 2.5% glutaraldehyde for transmission electron microscopy to examine lysosomal ultrastructure.

### 2.8 Statistical Analysis

Descriptive statistics were used to summarize the general characteristics of the study population. Categorical variables were expressed as frequencies and percentages. The chi‐square test was employed to compare the rates of genetic diagnoses across different dystonia subtypes. Statistical analyses were performed using SPSS software (version 22.0), with *p* < 0.05 considered statistically significant.

## 3 Results

### 3.1 Genetic Architecture of Dystonia in the Chinese Cohort

A total of 147 dystonia patients (75 males and 72 females) were enrolled, with a mean age of 34.2 ± 17.1 years and a mean age at onset of 30.3 ± 19.9 years. According to age at onset, 48 patients (32.7%) had childhood onset (2–12 years), 22 (15.0%) adolescent onset (13–18 years), 44 (29.9%) early adulthood onset (19–40 years), and 33 (22.4%) late adulthood onset (> 40 years). No patients had infancy onset (< 2 years). Based on anatomical distribution, 33 patients (22.4%) had generalized dystonia, 35 (23.8%) multifocal dystonia, 62 (42.2%) focal dystonia, and 17 (11.6%) segmental dystonia. No patients were classified as having hemidystonia. A positive family history was present in 19 patients (12.9%) (Table 1).

**TABLE 1:** Demographic and clinical characteristics of dystonia patients in this study.

Characteristics	Total number of probands (*N* = 147)
Gender	
Men	75
Women	72
Age	
0–12 (Infancy to childhood)	5
13–18 (Adolescence)	33
> 19 (Adulthood)	109
Age of onset	
0–2 (Infancy)	/
2–12 (Infancy to childhood)	48
13–18 (Adolescence)	22
19–40 (Early adulthood)	44
> 41 (Late adulthood)	33
Clinical presentation	
Generalized	33
Multifocal	35
Focal	62
Segmental	17
Hemidystonia	/
Additional clinical characteristics	
Speech disorder	35
Intellectual disability	16
Ataxia	6
Myoclonus	2
Parkinsonism	12
Brain MRI abnormality	6
Family history	19

Abbreviation: MRI, magnetic resonance imaging.

At the variant level, a total of 83 rare variants were identified in the cohort, including 47 missense, five nonsense, 11 frameshift, nine small insertions/deletions, four splice‐site variants, and seven copy‐number variants. Among these, 38 variants were classified as P/LP, whereas 45 were variants of uncertain significance (VUS) (Figure 1A, Table S1).

**FIGURE 1:**
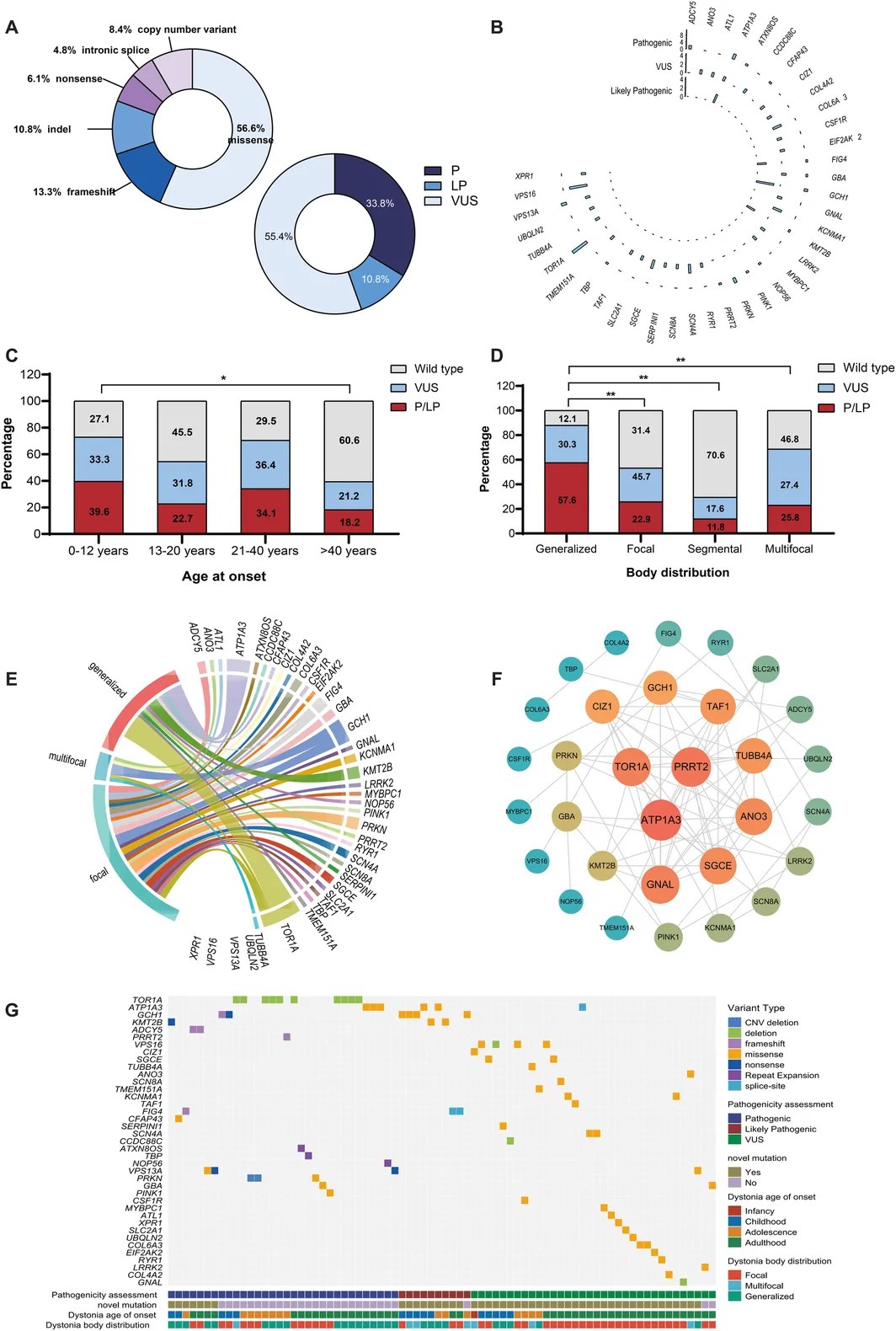
Genetic landscape of dystonia in a Mainland Chinese cohort. (A) Classification of detected variant types and ACMG pathogenicity ratings. LP, likely pathogenic; P, pathogenic; VUS, variants of uncertain significance. (B) Distribution of patient numbers carrying mutations in different genes (bell‐shaped plot). (C) Diagnostic yield was higher in childhood‐onset cases compared with adult‐onset cases (39.6% vs. 18.2%, *p < 0.05). (D) The generalized dystonia subgroup showed a significantly higher diagnostic yield than the focal, segmental, and multifocal subtypes (57.6% vs. 22.9%, 11.8%, and 25.8%, **p < 0.01). (E) Chord diagram showing the associations between mutated genes and dystonia phenotypes (focal, multifocal, and generalized). (F) Network diagram illustrating the interactions among dystonia‐associated genes. (G) Mutation spectrum of 147 Chinese dystonia patients, displaying variant types and pathogenicity per patient, along with clinical characteristics including age of onset and dystonia subtype.

Overall, molecular diagnosis was achieved in 49 of 147 patients, yielding a diagnostic yield of 33.3%. Subgroup analysis demonstrated that childhood‐onset cases exhibited a higher diagnostic rate than adult‐onset cases (39.6% vs. 18.2%, *p* < 0.05). Likewise, patients with generalized dystonia had a significantly higher diagnostic yield than those with focal, segmental, or multifocal dystonia (57.6% vs. 22.9%, 11.8%, and 25.8%, respectively; *p* < 0.01) (Figure 1C,D).

In our cohort, a limited number of recurrent genes, including *TOR1A*, *GCH1*, *ATP1A3*, and *KMT2B*, accounted for a substantial proportion of molecular diagnoses (Figure 1B). Variants in these core genes were mainly observed in carriers within specific age‐of‐onset groups and dystonia subtypes, with most classified as pathogenic or likely pathogenic. Notably, some core and other related genes were implicated across multiple dystonia phenotypes, encompassing focal, multifocal, and generalized forms. Network analysis further indicated that *TOR1A*, *GCH1*, *ATP1A3*, *PRRT2*, and *GNAL*, representing core and cross‐phenotype genes, occupy central positions within the dystonia gene interaction network, suggesting they may serve as key nodes in the genetic architecture of dystonia (Figure 1E–G).

### 3.2 Expansion of the Genetic and Phenotypic Spectrum in Dystonia

Our study identified two DYT‐*TOR1A* families, comprising 10 patients who carried the classical *TOR1A* c.907_909delGAG (p.E303del) (Figure S1). The age of onset ranged from 7 to 50 years, with initial symptoms primarily manifesting in the limbs and neck. Patients with limb‐onset dystonia presented with an earlier age of onset. The dystonic symptoms varied among these patients, including generalized, segmental, and focal types. The upper limbs were the most commonly affected, followed by the neck, lower limbs, and face. Two patients also exhibited psychiatric symptoms, previously diagnosed as paranoid psychosis, characterized by persecutory delusions and suicidal ideation.

We identified one family with DYT/PARK‐*ATP1A3*, in which three affected individuals carried the *ATP1A3* c.1838C>T (p.T613M) variant. Sanger sequencing confirmed segregation of these variants within the pedigree. Moreover, two novel *ATP1A3* missense variants, c.1103C>T (p.T368I) and c.2534G>C (p.G845A), were identified in two sporadic cases. Structural modeling showed that the novel mutations localize to core functional domains of *ATP1A3*, potentially affecting protein activity. CADD and minor‐allele‐frequency analyses indicated likely deleterious effects, while cross‐species conservation further highlighted the functional importance of these residues (Figure 2A–G).

**FIGURE 2:**
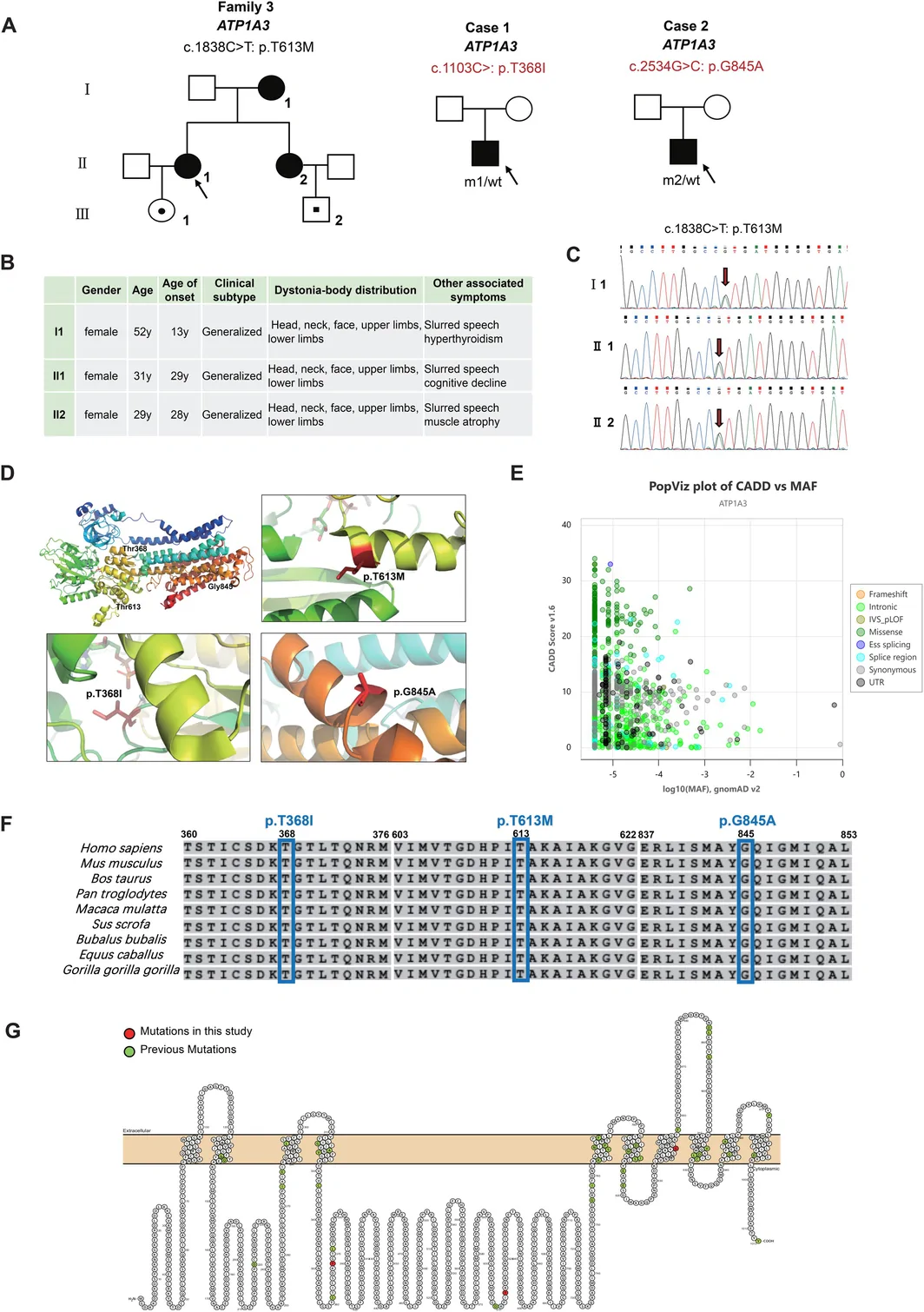
DYT/PARK‐*ATP1A3* pedigree and novel *ATP1A3* variants. (A) Pedigree of a family with DYT/PARK‐*ATP1A3*. Affected individuals are denoted by solid symbols. The pathogenic variant c.1838C>T (p.Thr613Met) and two putative novel mutations (Case 1/m1 c.1103C>T, p.Thr368Ile; Case 2/m2 c.2534G>C, p.Gly845Ala) are indicated. Novel mutations are highlighted in red. (B) Clinical phenotypes in the DYT/PARK‐*ATP1A3* pedigree. (C) Sanger sequencing results of patients from the DYT/PARK‐*ATP1A3* pedigree. (D) Three‐dimensional structural visualization of the novel mutations within the core functional domains of the *ATP1A3* protein. (E) CADD versus MAF plot of *ATP1A3* by PopViz. The horizontal and vertical axis shows MAF and CADD scores, respectively. (F) Conservation of amino acids at *ATP1A3* mutation sites across species. (G) Schematic representation of the *ATP1A3* transmembrane domains. Previously reported mutations are shown in green, and novel mutations from this study are labeled in red. Schematic representation of mutation sites was adapted from the Protter website.

In six patients with GCH1‐related dopa‐responsive dystonia (DYT/PARK‐*GCH1*), we identified several missense mutations in the *GCH1* gene, including c.424G>A (p.E142K), c.322G>T (p.G108C), c.335C>T (p.T112I), c.439C>T (p.P147S), and c.523T>G (p.Y175D). Additionally, two known hotspot mutations, c.259del (p.Q87Sfs*31) and c.562C>T (p.Q188*), were also detected. Structural modeling using AlphaFold and PyMOL showed that these novel mutations localize within the catalytic domain, potentially affecting enzyme function or protein stability. CADD analysis indicated that these variants are likely deleterious (Figure 3A–E).

**FIGURE 3:**
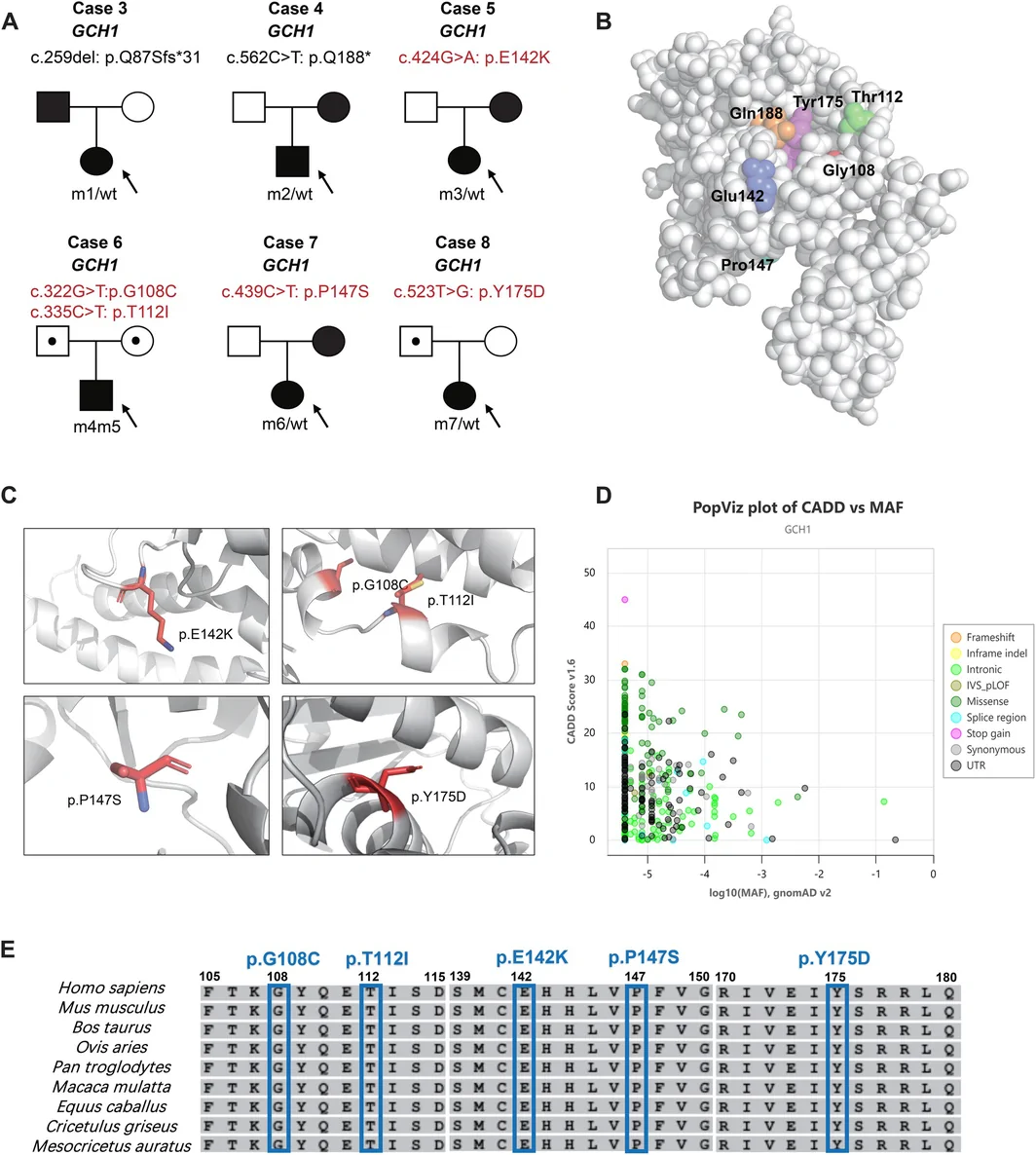
Identification of novel *GCH1* variants in sporadic dystonia. (A) Sporadic cases carrying *GCH1* mutations. The variants marked in red represent novel mutations that have not been previously reported. (B) Three‐dimensional structural visualization of the novel *GCH1* mutations by AlphaFold software, showing distribution of mutated amino acids. (C) PyMOL visualization of specific mutation sites. (D) CADD versus MAF plot of *GCH1* by PopViz. The horizontal and vertical axis shows MAF and CADD scores, respectively. (E) Conservation of amino acids at *GCH1* mutation sites across species.

We identified three novel *KMT2B* variants (c.3611G>A, p.C1204Y; c.889C>T, p.R297X; c.3720T>G, p.H1240Q) in three patients with DYT‐*KMT2B* (Figure S2). Case 9, a 15‐year‐old female, presented with cervical dystonia progressing to the trunk and lower limbs, accompanied by torsional posturing, orofacial dyskinesia, and dysarthria, showing a segmental‐to‐generalized pattern. Case 10 exhibited adolescent‐onset lower limb dystonia that gradually generalized, consistent with typical *KMT2B*‐related features. Notably, Case 11 showed paroxysmal dystonia superimposed on generalized dystonia, expanding the phenotypic spectrum associated with *KMT2B*. We also found the previously reported *PRRT2* mutation (c.649dupC, p.R217Pfs*8) in an 18‐year‐old male with typical paroxysmal kinesigenic dyskinesia (PKD).

Additionally, we identified VUS in genes previously linked to dystonia, including *ADCY5, VPS16*, *CIZ1*, *SGCE*, *TUBB4A*, *ANO3*, *SCN8A*, *TMEM151A*, *KCNMA1*, and *TAF1*. While CADD and REVEL scores suggest these variants may be deleterious, further evidence is needed to establish their pathogenicity (Table S1). In addition, several patients carried VUS in other genes, including *SCN4A*, *SERPINI1*, and *CCDC88C*, associated with focal or generalized dystonia, and their contribution to dystonia requires further functional validation (Table S2).

### 3.3 Dystonia Presentations in Nontraditional Dystonia Genes

We identified two heterozygous *FIG4* variants in three patients presenting with dystonia‐predominant phenotypes. Case 28, an 18‐year‐old female with paroxysmal dystonia, carried a frameshift mutation (c.1038_1039del, p.L347Gfs*15). Case 29, a 46‐year‐old female with focal cervical dystonia, harbored a splice‐site variant (c.2181‐2A>G) that was also present in her affected sister, demonstrating intrafamilial phenotypic variability. Notably, their mother also carried the same heterozygous variant but was asymptomatic, indicating incomplete segregation and limiting causal interpretation (Figure 4A–H). Minigene analysis of c.2181‐2A>G demonstrated aberrant splice‐site usage with retention of one terminal intronic G (Figure S3). Exploratory functional studies in HEK‐293 T cells showed lysosome‐related alterations in cells expressing the *FIG4* MUT constructs, including changes in LysoTracker‐positive compartments, lysosomal morphology, autophagy‐related markers, and cell viability (Figure S4).

**FIGURE 4:**
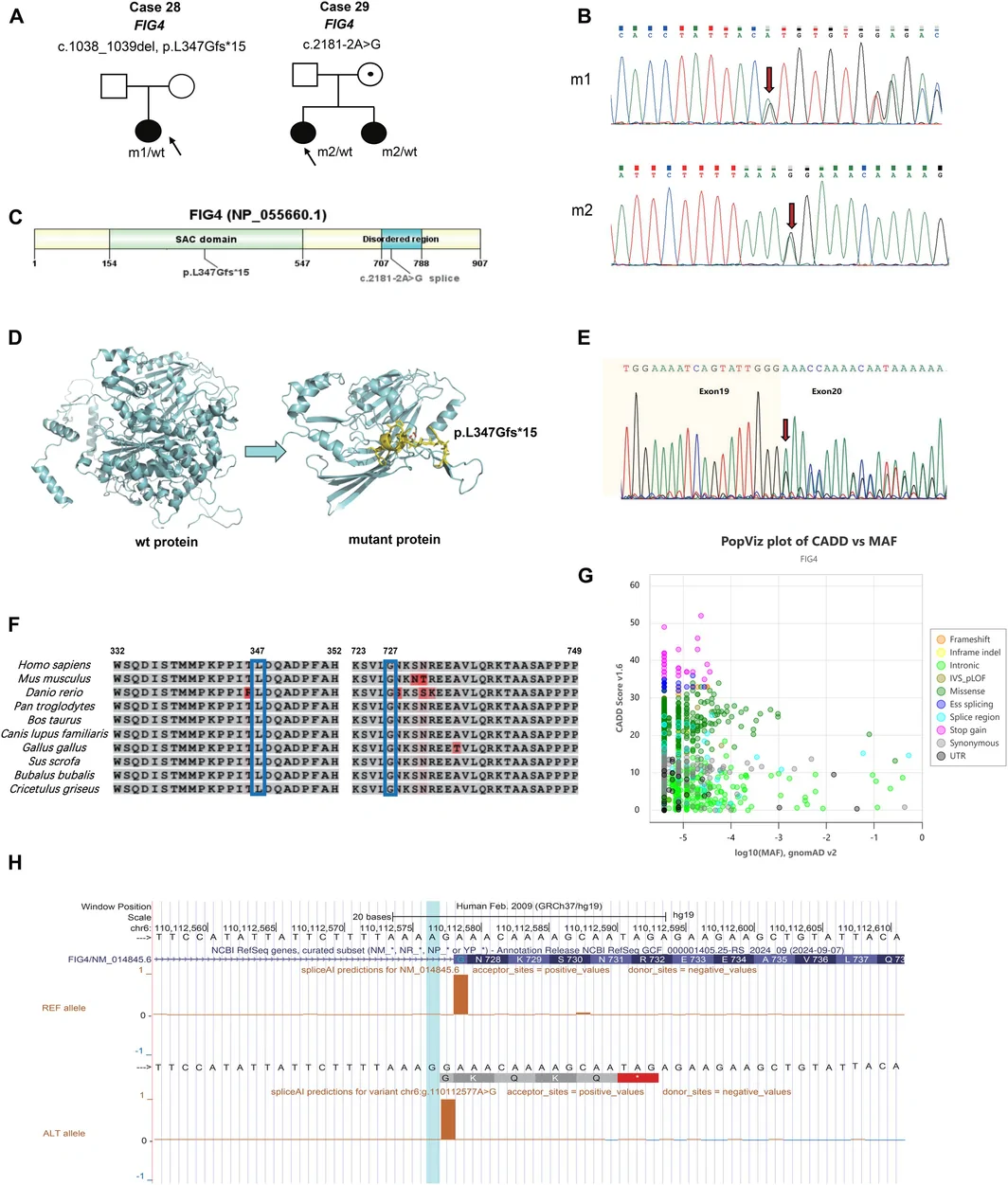
Candidate *FIG4* variants identified in patients with dystonia‐predominant phenotypes. (A) Cases carrying *FIG4* gene variants: Case 28 (c.1038_1039del) and Case 29 (c.2181‐2A>G). (B) Sanger sequencing validation of the *FIG4* variants. (C) Schematic representation of the *FIG4* gene structure. The c.1038_1039del mutation is located in the SAC domain, while c.2181‐2A>G is located in the disordered region. (D) Three‐dimensional structural visualization of the c.1038_1039del (p.L347Gfs*15) mutation. (E) Sanger sequencing of amplified cDNA from the Case 29 patient's blood RNA confirmed the c.2181‐2A>G splicing variant at the canonical acceptor site. (F) Conservation of amino acids at *FIG4* mutation sites across species. (G) CADD versus MAF plot of *FIG4* by PopViz. The horizontal and vertical axes show MAF and CADD scores, respectively. (H) Splice‐AI predicts that the c.2181‐2A>G variant will introduce a premature termination codon, likely triggering nonsense‐mediated mRNA decay and resulting in the loss of functional protein.

Repeat expansion analysis identified two repeat expansions within previously reported disease‐associated or incompletely penetrant ranges, but both were interpreted as being of uncertain clinical significance in the present clinical context. Case 35 was a 28‐year‐old male with focal right upper limb dystonia, characterized by sustained elevation of the right arm and clumsy handwriting. Genetic testing identified 119 CTA·TAG repeats in the *ATXN8OS* gene, with a previously reported range for SCA8 (Figure S5). Case 36 was a 33‐year‐old female with a 14‐month history of cervical dystonia presenting as lateral neck deviation, predominantly to the left, with botulinum toxin therapy achieving up to 90% symptomatic improvement. She harbored 42 CAG repeats in the *TBP* gene, corresponding to the incompletely penetrant range for SCA17. Comprehensive neurological examination revealed no evidence of cerebellar dysfunction in either patient, with normal SARA scores, preserved tandem gait, and negative Romberg sign. Accordingly, these repeat expansion findings were not considered definitive molecular diagnoses.

Some patients carried variants in Parkinson's disease‐associated genes, including *PRKN*, *PINK1*, and *GBA* (Table S2). Among these, Case 43 harbored a homozygous *PRKN* deletion and presented with a dystonia‐predominant movement disorder (Figure S6). The remaining patients carried heterozygous or likely non‐causative variants in *PINK1* and *GBA*. Two patients carrying homozygous *VPS13A* mutations were reclassified as having neuroacanthocytosis, despite presenting with dystonia‐like phenotypes. These findings illustrate the phenotypic variability of Parkinson's disease‐related genes and support the value of genetic testing in atypical movement disorder presentations.

## 4 Discussion

This study characterized the genetic landscape of dystonia in a Mainland Chinese cohort using WES, with repeat‐expansion screening used as a complementary analysis for selected phenocopy evaluation. This approach expanded the mutational spectrum and associated phenotypic range of established dystonia‐related genes, including *ATP1A3*, *KMT2B*, *GCH1*, and *TOR1A*. In addition, we observed heterozygous *FIG4* variants in three patients; however, these findings should be interpreted as candidate observations, and their disease relevance requires further validation. Overall, these results contribute to refining the molecular classification of dystonia and provide a cautious foundation for future functional studies and genotype‐informed therapeutic exploration.

While the overall diagnostic yield of WES in our cohort was comparable to Western reports (20%–33%) [11], we observed distinct differences in the distribution of causative genes. Unlike Western populations, where adult‐onset focal/segmental dystonia predominates (primarily associated with *TOR1A*, *THAP1*, and *GNAL* variants [26, 27]), our cohort exhibited a higher prevalence of childhood‐onset generalized dystonia (47.7%), and childhood‐onset cases demonstrated a significantly higher diagnostic yield than adult‐onset cases (39.6% vs. 18.2%), with *KMT2B*, *ATP1A3*, and *TOR1A* being the top causative genes in our cohort. These distinct genetic patterns reflected population‐specific genetic architectures, suggesting that East Asian populations exhibited characteristic dystonia genetic profiles. These findings collectively emphasized the need for ethnicity‐specific genomic databases, customized gene panels, and diagnostic workflows that integrated clinical phenotype, onset age, and population‐specific genetic factors to optimize dystonia diagnosis [28].

Previous studies had highlighted the marked clinical heterogeneity of *KMT2B*‐related dystonia [29]. In our cohort, two DYT‐*KMT2B* cases further exemplified this variability: Case 9 displayed atypical cervical‐onset dystonia that progressed to the upper limbs, deviating from the classic lower‐limb onset, while Case 11 presented paroxysmal dystonia, representing the first documented association of a *KMT2B* mutation with an episodic phenotype. In addition, our study demonstrated co‐occurrence of neuropsychiatric symptoms (including paranoid delusions and suicidal ideation) in DYT‐*TOR1A* and DYT/PARK‐*ATP1A3* families [30, 31]. Notably, this represents the first documentation of neuropsychiatric manifestations in patients with DYT/PARK‐*ATP1A3*. Overall, these findings underscore the imperative for implementing long‐term phenotypic monitoring and standardized psychiatric screening in atypical dystonia, while advocating for multidisciplinary team‐based approaches to optimize comprehensive patient management.

Repeat expansion screening identified *ATXN8OS* and *TBP* repeat expansions in two patients with dystonia‐dominant presentations but without cerebellar signs. Given the incomplete penetrance and interpretive complexity of these loci, these findings should be regarded as repeat expansions of uncertain clinical significance rather than definitive diagnoses of SCA8 or SCA17. These cases underscore that repeat‐expansion screening may provide complementary information in selected atypical movement disorder presentations, but genotype–phenotype attribution requires careful clinical correlation and should not rely on repeat size alone.

*FIG4* encodes a phosphoinositide phosphatase that participates in PI(3,5)P2 homeostasis and endolysosomal membrane trafficking. Pathogenic FIG4 variants have been implicated in several neurological disorders, including Yunis–Varón syndrome, Charcot–Marie–Tooth disease Type 4J, and amyotrophic lateral sclerosis [32, 33, 34, 35]. A recent report also described *FIG4*‐related dystonia‐parkinsonism associated with a homozygous variant, suggesting that *FIG4* dysfunction may be associated with movement disorder phenotypes [36]. In the present cohort, we identified heterozygous *FIG4* variants in patients with dystonia‐predominant presentations. Although these findings do not establish *FIG4* as a definitive dystonia‐causing gene, they represent candidate observations worthy of further investigation. This possibility is biologically plausible given the established role of *FIG4* in lysosome‐related pathways. *FIG4* functions within a regulatory complex with VAC14 and PIKfyve to maintain PI(3,5)P2 homeostasis, which is important for endolysosomal membrane dynamics and intracellular trafficking [37]. Consistent with this pathway, our exploratory cellular analyses suggested that *FIG4* perturbation may influence lysosome‐related cellular homeostasis. These findings are also compatible with previous evidence that *FIG4* deficiency can affect nervous system function and motor phenotypes in animal models [38].

This study has several limitations. First, the *FIG4* findings should be interpreted as candidate observations, and their disease relevance remains to be established in neuronal models, patient‐derived cells, or animal models. In particular, the functional experiments were performed in HEK‐293T cells, and the biological relevance of lysosome‐related cellular changes to dystonia requires further validation. Second, the cohort size was modest, and larger independent cohorts with case–control burden analyses are required to validate candidate genes and refine genotype–phenotype correlations. Third, segregation analysis was incomplete in some families because of limited sample availability and follow‐up. Finally, WES does not fully capture non‐coding variants, deep intronic variants, regulatory variants, mitochondrial variants, or large and complex structural variants. Recent work by Zech et al. highlighted the potential value of whole‐genome sequencing and fibroblast‐derived proteomics for further molecular diagnosis and variant interpretation in WES‐unresolved dystonia [39]. Future studies incorporating WGS and functional multi‐omic approaches may therefore help resolve genetically unexplained cases and provide a more comprehensive view of dystonia genetics in East Asian populations.

## 5 Conclusion

This study delineates the genetic architecture and genotype–phenotype spectrum of dystonia in a Chinese cohort, providing population‐specific evidence for the molecular classification and genetic evaluation of dystonia.

## Author Contributions

**Yiqing Gong:** conceptualization, methodology, investigation, formal analysis, writing – original draft, data curation. **Huirong Peng:** investigation, formal analysis. **Zhao Chen:** data curation, formal analysis. **Jiawei He:** methodology, formal analysis. **Linliu Peng:** methodology, investigation. **Qian Jiang:** software. **Chunrong Wang:** investigation. **Linlin Wan:** investigation. **Lang He:** investigation. **Yuting Shi:** investigation. **Rong Qiu:** writing – review and editing. **Hong Jiang:** conceptualization, methodology, investigation, writing‐original draft, writing – review and editing, supervision, project administration.

## Funding

This study was funded by the National Key R&D Program of China (No. 2021YFA0805200 to H. Jiang), the National Natural Science Foundation of China (U25A2058, 82571433 to H. Jiang; 82571632, 82371272 to Z. Chen; 82301628 to L. Wan; 82401496 to L. Peng), the Scientific Research Program of FuRong Laboratory (2025PT5014, 2025PT5001 to H.Jiang), the Natural Science Foundation of Hunan Province (No. 2024JJ3050 to H. Jiang; 2025JJ20078 to Z. Chen; 2024JJ6638 to L. Wan; 2025JJ60489 to L. Peng), The Science and Technology Innovation Program of Hunan Province (2026RC9017 to H. Jiang), the Major Scientific Research Project for High‐Level Health Talent in Hunan Province (No. R2023047 to H. Jiang), MIIT Project for Open Competition and leadership in AI Medical Device Innovation (2025‐AISJK‐01009 to H Jiang), the Central South University Research Programme of Advanced Interdisciplinary Study (No. 2023QYJC010 to H. Jiang), Central South University Clinical Research Zhang Xiao‐qian Program (ZXQ2026A08 to H Jiang), Postdoctoral Fellowship Program of CPSF (GZB20230870 to L. Peng), and China Postdoctoral Science Foundation (2024M753690 to L. Peng).

## Conflicts of Interest

The authors declare no conflicts of interest.

## Supporting information


**Figure S1:** Genetic and clinical characterization of two DYT‐*TOR1A* families. (A) Pedigrees of Family 1 and Family 2 carrying the pathogenic *TOR1A* variant (c.907_909delGAG, p.E303del). (B) Affected individuals exhibit marked variability in age at onset and clinical manifestations. In Family 1, the proband (F1‐I1, female, 77 years) showed cranial–cervical and limb dystonia accompanied by neuropsychiatric symptoms, while her son (F1‐II2, male, 53 years) presented with generalized dystonia and psychiatric features. Other relatives (F1‐II3 and F1‐II4) displayed focal dystonia involving the head/neck or upper limbs. In Family 2, the proband (F2‐II11, male, 32 years) had generalized dystonia with dysarthria and cognitive impairment, whereas an asymptomatic carrier (F2‐II12, female, 6 years) was identified, consistent with incomplete penetrance. Across both families, age at onset ranged from 7 to 52 years, and disease distribution varied from focal to generalized dystonia.
**Figure S2:** Genotype–phenotype correlations in dystonia patients with three novel *KMT2B* variants. (A) Three previously unreported *KMT2B* variants were identified in three unrelated patients. Case9 (m1), c.3611G>A (p.C1204Y); Case10 (m2), c.889C>T (p.R297*); Case11 (m3), c.3720T>G (p.H1240Q). (B) Representative clinical phenotypes of the three patients. Case 9 presented with adolescent‐onset cervical dystonia that progressed to generalized dystonia with truncal and orofacial involvement, including blepharospasm and dysarthria. Case 10 exhibited a typical *KMT2B*‐related dystonia phenotype with adolescent‐onset lower limb dystonia that gradually generalized. Case 11 showed a distinctive phenotype of generalized dystonia with superimposed paroxysmal dystonic attacks affecting the right upper and lower limbs, characterized by activity‐induced exacerbation and complete resolution during sleep, representing a previously unreported *KMT2B*‐associated presentation. (C) CADD versus MAF plot of *KMT2B* by PopViz. The horizontal and vertical axis shows MAF and CADD scores, respectively.
**Figure S3:** Minigene analysis of the *FIG4* c.2181‐2A>G splice‐acceptor variant. (A) Schematic representation of the splicing consequence of the *FIG4* c.2181‐2A>G variant. In the WT construct, the canonical AG splice acceptor at the 3′ end of the intron upstream of exon 20 is normally recognized, resulting in correct joining of exons 19 and 20. The Gly727 codon (GGA) spans the exon 19–exon 20 junction, with the first two G nucleotides located at the terminal sequence of exon 19 and the third nucleotide, A, corresponding to the first coding nucleotide of exon 20. In the mutant construct, c.2181‐2A>G disrupts the canonical splice acceptor and promotes the use of an upstream cryptic AG acceptor located 1 bp upstream. Consequently, the splice junction shifts 1 bp upstream, resulting in retention of the terminal intronic G in the mature transcript. This retained nucleotide changes the Gly727 codon from GGA to GGG without altering the encoded amino acid, but shifts the downstream reading frame beginning at codon 728 and introduces a premature termination codon within exon 20. The predicted protein consequence is p.N728Kfs*5. Blue indicates exonic sequence, black indicates intronic sequence, and red indicates the substituted nucleotide and the retained terminal intronic G. (B) Representative Sanger sequencing chromatograms of minigene‐derived WT and c.2181‐2A>G mutant transcripts. The WT transcript shows normal exon 19–exon 20 joining, whereas the mutant transcript shows retention of one terminal intronic G at the splice junction, consistent with use of the upstream cryptic splice acceptor. The retained intronic G is highlighted by the red box and arrow.
**Figure S4:** Exploratory analysis of lysosome‐related cellular phenotypes in HEK‐293T cells expressing *FIG4* variants. (A) Anti‐FLAG immunoblotting of cells expressing wild‐type (WT) *FIG4* and the indicated truncated *FIG4* variants. m1 represents c.1038_1039del, predicted to result in p.L347Gfs*15, whereas m2 represents the truncated coding product derived from the aberrantly spliced c.2181‐2A>G transcript, p.N728Kfs*5. The m1 and m2 proteins migrated at approximately 40 kDa and 85 kDa, respectively, compared with the full‐length WT *FIG4* protein at approximately 100 kDa. (B) Representative immunoblots of the lysosome–and autophagy‐related proteins LAMP1, p62, LC3‐I, and LC3‐II in cells expressing WT, m1, or m2. GAPDH was used as a loading control. (C) Quantification of LAMP1 and p62 protein levels and the LC3‐II/LC3‐I ratio. (D) Cell viability assessed by the CCK‐8 assay and expressed as absorbance at 450 nm. (E) Representative flow‐cytometric profiles of LysoTracker fluorescence in cells expressing WT, m1, or m2. (F) Quantification of LysoTracker mean fluorescence intensity (MFI). (G) Representative fluorescence images showing LysoTracker staining (red) and Hoechst nuclear staining (blue) in the indicated groups. (H) Quantification of LysoTracker fluorescence intensity. (I) Representative transmission electron microscopy images showing lysosome‐related ultrastructural changes in cells expressing WT, m1, or m2. (J) Quantification of lysosome numbers in the indicated groups. Data are presented as mean ± SEM. ns, not significant; *p < 0.05, **p < 0.01, and ***p < 0.001.
**Figure S5:** Detection of SCA‐related repeat expansions. Capillary electrophoresis confirmed repeat expansions in ATXN8OS (Case 35) and TBP (Case 36). Both findings were considered of uncertain clinical significance in the present clinical context. Clinical features and repeat sizes: Case 35 showed focal right upper limb dystonia without ataxia, carrying 29/>119 CTA·TAG repeats in *ATXN8OS*. Case 36 exhibited cervical dystonia responsive to botulinum toxin and carried 42 CAG repeats in *TBP* (incompletely penetrant SCA17 range).
**Figure S6:** Detection of *PRKN* (*PARK2*) copy number variations. Multiplex ligation‐dependent probe amplification (MLPA) identified homozygous deletions in PRKN (exon 2 in Case 43). The red mark indicates the deletion.

**Table S1:** Genotypes, phenotypic features, and ACMG classification of dystoniaassociated variants.

**Table S2:** Variants in genes associated with dystonia‐related differential diagnoses.

## Data Availability

The data that support the findings of this study are available from the corresponding author upon request. The variation data reported in this paper have been deposited in the Genome Variation Map (GVM) at the National Genomics Data Center, Beijing Institute of Genomics, Chinese Academy of Sciences, and the China National Center for Bioinformation, under accession number GVM001195.
