# Supplementary material for: Whole‐Exome Sequencing Reveals the Genetic Landscape and Genotype–Phenotype Spectrum of Dystonia in a Chinese Cohort
Source: CNS Neurosci Ther. 2026 Oct 11;32(10):e71203. doi: 10.1002/cns.71203 (PMC13655300; doi:10.1002/cns.71203)
Supplement: Supplementary file 1 [file CNS-32-e71203-s001.docx]

**Supplementary Table 1.** **Genotypes, phenotypic features, and ACMG classification of dystonia-associated variants**

| **Gene** | **Patients** | **cDNA variants** | **Predicted protein changes** | **Variant type** | **Variant inheritance**  **/Zygosity** | **CADD**  **/Revel** | **ACMG evidence** | **ACMG** | **novel**  **(yes/no)** | **Phenotype**  **(onset/distribution)** |
| --- | --- | --- | --- | --- | --- | --- | --- | --- | --- | --- |
| *TOR1A* | Family 1, Family 2 | c.907_909delGAG | p.E303del | deletion | AD/het | / | PS3^a^, PS4^a^ | Pathogenic | no | adulthood/generalized |
| *ATP1A3* | Family 3 | c.1838C>T | p.T613M | missense | AD/het | 33/0.99 | PS3^b^, PS4^b^, PP1, PP3 | Pathogenic | no | adulthood/generalized |
| *ATP1A3* | Case 1 | c.1103C>T | p.T368I | missense | AD/het | 28.5/0.99 | PM1, PM2, PM6, PP2, PP3 | Likely Pathogenic | no | childhood/generalized |
| *ATP1A3* | Case 2 | c.2534G>C | p.G845A | missense | AD/het | 24/0.79 | PS2, PM2, PP1, PP3 | Likely Pathogenic | no | adolescence/generalized |
| *GCH1* | Case 3 | c.259del | p.Q87Sfs*31 | frameshift | AD/het | / | PVS1, PM2, PP4 | Pathogenic | no | childhood/focal |
| *GCH1* | Case 4 | c.562C>T | p.Q188* | stop-gain | AD/het | 44/- | PVS1, PM2, PP4 | Pathogenic | no | childhood/focal |
| *GCH1* | Case 5 | c.424G>A | p.E142K | missense | AD/het | 33 | PM1, PM2, PP3, PP4 | Likely Pathogenic | yes | childhood/focal |
| *GCH1* | Case 6 | c.322G>T | p.G108C | missense | AD/het | 33/0.99 | PM1, PM2, PP2, PP3 | Likely Pathogenic | yes | childhood/multifocal |
| *GCH1* | Case 6 | c.335C>T | p.T112I | missense | AD/het | 24.5/0.83 | PM1, PM2, PP2, PP3 | Likely Pathogenic | yes | childhood/multifocal |
| *GCH1* | Case 7 | c.439C>T | p.P147S | missense | AD/het | 30/0.99 | PM2, PM5^a^, PP2, PP3 | Likely Pathogenic | yes | childhood/focal |
| *GCH1* | Case 8 | c.523T>G | p.Y175D | missense | AD/het | 32/1.0 | PM2, PM5^b^, PP2, PP3 | Likely Pathogenic | no | adolescence/multifocal |
| *KMT2B* | Case 9 | c.3611G>A | p.C1204Y | missense | AD/het | 26.6/0.98 | PS2, PM2, PP1, PP3 | Likely Pathogenic | yes | adolescence/generalized |
| *KMT2B* | Case 10 | c.889C>T | p.R297* | stop-gain | AD/het | 36/- | PVS1, PM2, PP1, PP4 | Pathogenic | yes | childhood/generalized |
| *KMT2B* | Case 11 | c.3720T>G | p.H1240Q | missense | AD/het | 13.5/0.75 | PM2, PP1, PP3 | VUS | yes | childhood/generalized |
| *ADCY5* | Case 12 | c.556delG | p.D186Mfs*52 | frameshift | AD/het | / | PM2, PP3 | VUS | yes | adulthood/focal |
| *ADCY5* | Case 13 | c.3143del | p.V1048Gfs*109 | frameshift | AD/het | / | PM2, PP3 | VUS | yes | adulthood/focal |
| *PRRT2* | Case 14 | c.649dupC | p.R217Pfs*8 | frameshift | AD/het | 35/- | PVS1, PM2, PP3 | Pathogenic | no | adolescence/generalized |
| *VPS16* | Case 15 | c.425C>T | p.S142F | missense | AD/het | 26.7/0.52 | PM2, PP3 | VUS | yes | childhood/focal |
| *VPS16* | Case 16 | c.1763_1765  del | p.G588del | deletion | AD/het | / | PM2, PP3 | VUS | yes | childhood/generalized |
| *VPS16* | Case 17 | c.1591C>T | p.R531C | missense | AD/het | 32/0.83 | PM2, PP3 | VUS | yes | adulthood/focal |
| *VPS16* | Case 18 | c.493C>T | p.R165C | missense | AD/het | 32/0.7 | PM2, PP3 | VUS | yes | adolescence/focal |
| *CIZ1* | Case 19 | c.2642C>T | p.T881M | missense | AD/het | 23/0.38 | PM2, PP3 | VUS | yes | Infancy/multifocal |
| *SGCE* | Case 20 | c.143G>A | p.R48Q | missense | AD/het | 32/0.84 | PM2, PP3 | VUS | yes | adulthood/focal |
| *SGCE* | Case 21 | c.1283T>C | p.L428S | missense | AD/het | 32/0.71 | PM2, PP3 | VUS | yes | childhood/focal |
| *TUBB4A* | Case 22 | c.1272G>C | p.Q424H | missense | AD/het | 25/0.77 | PM2, PP3 | VUS | yes | adolescence/multifocal |
| *ANO3* | Case 23 | c.149T>G | p.L50W | missense | AD/het | 25.2/0.37 | PM2, PP3 | VUS | yes | adulthood/multifocal |
| *SCN8A* | Case 24 | c.5747T>C | p.I1916T | missense | AD/het | 22.2/0.81 | PM2, PP3 | VUS | yes | adulthood/focal |
| *TMEM151A* | Case 25 | c.796T>A | p.F266I | missense | AD/het | 27.4/0.75 | PM2, PP3 | VUS | yes | adolescence/generalized |
| *KCNMA1* | Case 26 | c.3485A>G | p.Y1162C | missense | AD/het | 25.9/0.9 | PM2, PP3 | VUS | yes | adulthood/focal |
| *TAF1* | Case 27 | c.2299C>T | p.R787W | missense | XR/hem | 26.7/0.91 | PM2, PP3 | VUS | yes | adulthood/focal |

Abbreviations: OMIM, online Mendelian Inheritance in Man; AD, autosomal-dominant; AR, autosomal-recessive; hem, hemizygous; het, heterozygous; hom, homozygous; CADD: the score more than 20 is considered as deleteriousness for SNP; Revel: the score more than 0.65 is considered as deleteriousness for SNP; gnomAD, Genome Aggregation Database; VUS, variants of uncertain significance; PVS, very strong evidence of pathogenicity; PS, strong evidence of pathogenicity; PM, moderate evidence of pathogenicity; PP, supporting evidence of pathogenicity; /, not available.

ACMG criteria definitions:

PVS1: Null variant (nonsense, frameshift, canonical ±1 or 2 splice site) in a gene where loss of function is a known disease mechanism.

PS2: De novo variant (confirmed in patient and absent in both parents), supporting strong evidence of pathogenicity.

PS3^a^： Functional studies demonstrate that *TOR1A* p.E303del (ΔE) leads to abnormal subcellular localization and reduced ATPase activity of TorsinA protein, consistent with a damaging effect on protein function.

PS3^b^: The *ATP1A3* p.T613M variant leads to abnormal protein expression and impaired neuronal survival, supporting a deleterious effect on the gene product.

Reference: De Carvalho Aguiar P et al., Neurology, 2004; 63: 1777–1780, PMID:15260953.

PS4^a^： The p.E303del variant has been reported in multiple unrelated patients and families with early-onset generalized dystonia (Dystonia 1, OMIM 128100), indicating significant enrichment in affected individuals compared with population controls.

PS4^b^: The variant has been observed in multiple unrelated patients, with a frequency significantly higher in affected individuals than in the general population, supporting its association with disease.

PM1: Located in a mutational hotspot and/or critical and well-established functional domain without benign variation.

PM2 (PM2_supporting): Absent from population database gnomAD v2.1.0.

PM5^a^: Novel missense variant (p.P147S) occurs at the same amino acid residue (P147) as a previously established pathogenic missense variant p.P147Q, supporting moderate evidence of pathogenicity. Reference: Ichinose H et al., Neurology, 2009; 72: 1185–1191, PMID:19491146.

PM5^b^: Novel missense variant (p.Y175D) occurs at the same amino acid residue (Y175) as a previously established pathogenic missense variant p.Y175C, supporting moderate evidence of pathogenicity. Reference: Guella I et al., Am J Hum Genet, 2007; 81: 1161–1170, PMID:17898029.

PM6: Assumed de novo variant, but without confirmation of paternity and maternity.

PP3: Multiple computational tools predict a deleterious effect on the gene or gene product. In silico predictions were evaluated using REVEL and CADD.

PP4: Patient's phenotype or family history is highly specific for a disease with a single genetic etiology.
