# Supplementary material for: Whole‐Exome Sequencing Reveals the Genetic Landscape and Genotype–Phenotype Spectrum of Dystonia in a Chinese Cohort
Source: CNS Neurosci Ther. 2026 Oct 11;32(10):e71203. doi: 10.1002/cns.71203 (PMC13655300; doi:10.1002/cns.71203)
Supplement: Supplementary file 2 [file CNS-32-e71203-s002.docx]

**Supplementary Material**

**Supplementary Figure 1.** **Genetic and clinical characterization of two DYT-*TOR1A* families. (A)** Pedigrees of Family 1 and Family 2 carrying the pathogenic *TOR1A* variant (c.907_909delGAG, p.E303del). **(B)** Affected individuals exhibit marked variability in age at onset and clinical manifestations. In Family 1, the proband (F1-I1, female, 77 years) showed cranial–cervical and limb dystonia accompanied by neuropsychiatric symptoms, while her son (F1-II2, male, 53 years) presented with generalized dystonia and psychiatric features. Other relatives (F1-II3 and F1-II4) displayed focal dystonia involving the head/neck or upper limbs. In Family 2, the proband (F2-II11, male, 32 years) had generalized dystonia with dysarthria and cognitive impairment, whereas an asymptomatic carrier (F2-II12, female, 6 years) was identified, consistent with incomplete penetrance. Across both families, age at onset ranged from 7 to 52 years, and disease distribution varied from focal to generalized dystonia.

**Supplementary Figure 2. Genotype-phenotype correlations in dystonia patients with three novel *KMT2B* variants.** **(A)** Three previously unreported *KMT2B* variants were identified in three unrelated patients. Case9 (m1), c.3611G>A (p.C1204Y); Case10 (m2), c.889C>T (p.R297*); Case11 (m3), c.3720T>G (p.H1240Q). **(B)** Representative clinical phenotypes of the three patients. Case 9 presented with adolescent-onset cervical dystonia that progressed to generalized dystonia with truncal and orofacial involvement, including blepharospasm and dysarthria. Case 10 exhibited a typical *KMT2B*-related dystonia phenotype with adolescent-onset lower limb dystonia that gradually generalized. Case 11 showed a distinctive phenotype of generalized dystonia with superimposed paroxysmal dystonic attacks affecting the right upper and lower limbs, characterized by activity-induced exacerbation and complete resolution during sleep, representing a previously unreported *KMT2B*-associated presentation. **(C)** CADD vs MAF plot of *KMT2B* by PopViz. The horizontal and vertical axis shows MAF and CADD scores, respectively.

**Supplementary Figure 3.** **Minigene analysis of the *FIG4* c.2181-2A>G splice-acceptor variant. (A)** Schematic representation of the splicing consequence of the *FIG4* c.2181-2A>G variant. In the WT construct, the canonical AG splice acceptor at the 3′ end of the intron upstream of exon 20 is normally recognized, resulting in correct joining of exons 19 and 20. The Gly727 codon (GGA) spans the exon 19–exon 20 junction, with the first two G nucleotides located at the terminal sequence of exon 19 and the third nucleotide, A, corresponding to the first coding nucleotide of exon 20. In the mutant construct, c.2181-2A>G disrupts the canonical splice acceptor and promotes the use of an upstream cryptic AG acceptor located 1 bp upstream. Consequently, the splice junction shifts 1 bp upstream, resulting in retention of the terminal intronic G in the mature transcript. This retained nucleotide changes the Gly727 codon from GGA to GGG without altering the encoded amino acid, but shifts the downstream reading frame beginning at codon 728 and introduces a premature termination codon within exon 20. The predicted protein consequence is p.N728Kfs*5. Blue indicates exonic sequence, black indicates intronic sequence, and red indicates the substituted nucleotide and the retained terminal intronic G. **(B)** Representative Sanger sequencing chromatograms of minigene-derived WT and c.2181-2A>G mutant transcripts. The WT transcript shows normal exon 19–exon 20 joining, whereas the mutant transcript shows retention of one terminal intronic G at the splice junction, consistent with use of the upstream cryptic splice acceptor. The retained intronic G is highlighted by the red box and arrow.

**Supplementary Figure 4. Exploratory analysis of lysosome-related cellular phenotypes in HEK-293T cells expressing *FIG4* variants. (A)** Anti-FLAG immunoblotting of cells expressing wild-type (WT) *FIG4* and the indicated truncated *FIG4* variants. m1 represents c.1038_1039del, predicted to result in p.L347Gfs*15, whereas m2 represents the truncated coding product derived from the aberrantly spliced c.2181-2A>G transcript, p.N728Kfs*5. The m1 and m2 proteins migrated at approximately 40 kDa and 85 kDa, respectively, compared with the full-length WT *FIG4* protein at approximately 100 kDa. **(B)** Representative immunoblots of the lysosome- and autophagy-related proteins LAMP1, p62, LC3-I, and LC3-II in cells expressing WT, m1, or m2. GAPDH was used as a loading control. **(C)** Quantification of LAMP1 and p62 protein levels and the LC3-II/LC3-I ratio. **(D)** Cell viability assessed by the CCK-8 assay and expressed as absorbance at 450 nm. **(E)** Representative flow-cytometric profiles of LysoTracker fluorescence in cells expressing WT, m1, or m2. **(F)** Quantification of LysoTracker mean fluorescence intensity (MFI). **(G)** Representative fluorescence images showing LysoTracker staining (red) and Hoechst nuclear staining (blue) in the indicated groups. **(H)** Quantification of LysoTracker fluorescence intensity. **(I)** Representative transmission electron microscopy images showing lysosome-related ultrastructural changes in cells expressing WT, m1, or m2. **(J)** Quantification of lysosome numbers in the indicated groups. Data are presented as mean ± SEM. ns, not significant; **P* < 0.05, ***P* < 0.01, and ****P* < 0.001.

**Supplementary Figure 5. Detection of SCA-related repeat expansions**

Capillary electrophoresis confirmed repeat expansions in ATXN8OS (Case 35) and TBP (Case 36). Both findings were considered of uncertain clinical significance in the present clinical context. Clinical features and repeat sizes: Case 35 showed focal right upper limb dystonia without ataxia, carrying 29/>119 CTA·TAG repeats in *ATXN8OS*. Case 36 exhibited cervical dystonia responsive to botulinum toxin and carried 42 CAG repeats in *TBP* (incompletely penetrant SCA17 range).

**Supplementary Figure 6. Detection of *PRKN* (*PARK2*) copy number variations**

Multiplex ligation-dependent probe amplification (MLPA) identified homozygous deletions in PRKN (exon 2 in Case 43). The red mark indicates the deletion.
