# Supplementary material for: Whole‐Exome Sequencing Reveals the Genetic Landscape and Genotype–Phenotype Spectrum of Dystonia in a Chinese Cohort
Source: CNS Neurosci Ther. 2026 Oct 11;32(10):e71203. doi: 10.1002/cns.71203 (PMC13655300; doi:10.1002/cns.71203)
Supplement: Supplementary file 3 [file CNS-32-e71203-s003.docx]

**Supplementary Table 2.** **Variants in genes associated with dystonia-related differential diagnoses**

| **Patient(s)** | **Gene** | **cDNA variant(s)** | **Variant inheritance/Zygosity** | **Variant type** | **Frequency in gnomAD** | **CADD score** | **Revel score** | **ACMG** | **ACMG evidence** | **novel mutation** |
| --- | --- | --- | --- | --- | --- | --- | --- | --- | --- | --- |
| Case 28 | *FIG4* | c.1038_1039del | AD/het | frameshift deletion | not found | / | / | Pathogenic | PVS1+PS2+PM2 | yes |
| Case 29 | *FIG4* | c.2181-2A>G | AD/het | splice-site | not found | / | / | Likely Pathogenic | PVS1+PM2 | yes |
| Case 33 | *SERPINI1* | c.737A>G | AD/het | missense | not found | 26.70 | 0.98 | VUS | PM2+PP3 | yes |
| Case 34 | *SCN4A* | c.4324G>A | AR/hom | missense | not found | 26.80 | 0.97 | VUS | PM2+PP3 | yes |
| Case 34 | *SCN4A* | c.3682G>A | AR/hom | missense | not found | 27.80 | 0.98 | VUS | PM2+PP3 | yes |
| Case35 | *ATXN8OS* | (CTA·TAG)n(CTG·CAG)n>119 | AD/het | repeat expansion | not found | / | / | / | / | no |
| Case36 | *TBP* | (CAG)n 35/42 | AD/het | repeat expansion | not found | / | / | / | / | no |
| Case 37 | *CCDC88C* | c.5832_5849del | AD/het | deletion | not found | / | / | VUS | PM2+PM4 | yes |
| Case38 | *VPS13A* | c.269T>C | AR/hom | missense | not found | 25.50 | 0.94 | Likely Pathogenic | PM1+PM2+PM5+PP3 | yes |
| Case38 | *VPS13A* | c.7777A>G | AR/hom | missense | not found | 21.20 | 0.12 | VUS | PM2 | yes |
| Case39 | *VPS13A* | c.3358A>T | AR/hom | nonsense | not found | 39.00 | / | Pathogenic | PVS1+PM2+PM3 | no |
| Case39 | *VPS13A* | c.7867C>T | AR/hom | nonsense | not found | 37.00 | / | Pathogenic | PVS1+PM2+PM3 | yes |
| Case 40 | *CFAP43* | c.782G>A | AD/het | missense | not found | 40.00 | 0.75 | Likely Pathogenic | PVS1+ PM2 | yes |
| Case41 | *GBA* | c.1448T>C | AD/het | missense | not found | 25.30 | 0.96 | Likely Pathogenic | PS3^a^+ PM5^b^+PP3 | no |
| Case42 | *PINK1* | c.1040T>C | AR/hom | missense | not found | 27.60 | 0.94 | Pathogenic | PS3^c^+PM2+PM3+PP1+PP3 | no |
| Case43 | *PRKN* | deletion of exon 2 | AR/hom | CNV deletion | not found | / | / | / | / | no |

Abbreviations: AD, autosomal-dominant; AR, autosomal-recessive; het, heterozygous; hom, homozygous; CADD: the score more than 20 is considered as deleteriousness for SNP; Revel: the score more than 0.65 is considered as deleteriousness for SNP; PVS, very strong evidence of pathogenicity; PS, strong evidence of pathogenicity; PM, moderate evidence of pathogenicity; PP, supporting evidence of pathogenicity; /, not available; VUS, variants of uncertain significance; CNV, copy number variation.

ACMG criteria definitions:

PVS1: Null variant (nonsense, frameshift, canonical ±1 or 2 splice site) in a gene where loss of function (LoF) is an established disease mechanism.

PS2: De novo status was confirmed by Sanger sequencing in both parents.

PS3^a^: Well-established functional studies supportive of a damaging effect on the gene or gene product. Functional studies show that the p.L483P (L444P) variant significantly reduces glucocerebrosidase enzymatic activity; reference: Montfort M et al., Hum Mutat, 2004, PMID:15146461.

PS3^c^: Well-established functional studies supportive of a damaging effect on the gene or gene product. Functional studies show that the PINK1 p.L347P variant destabilizes the protein and leads to rapid degradation, consistent with loss-of-function; reference: Moriwaki Y et al., Neurosci Res, 2008, PMID:18359116.

PM1: Located in a mutational hotspot and/or critical and well-established functional domain without benign variation.

PM2 (PM2_supporting): Absent from population database gnomAD v2.1.0.

PM3: For recessive disorders, detected in trans with a pathogenic variant.

PM4: Protein length changes due to in-frame deletions/insertions in a non-repeat region or stop-loss variants.

PM5^a^: Novel missense variant at the same amino acid residue as a previously established pathogenic variant (L483R).

PP1: Co-segregation with disease in multiple affected family members.

PP3: Multiple computational tools predict a deleterious effect on the gene or gene product. In silico predictions were evaluated using REVEL and CADD.
